# Genome-Wide Analysis and Expression Profiles of Ethylene Signal Genes and Apetala2/Ethylene-Responsive Factors in Peanut (*Arachis hypogaea* L.)

**DOI:** 10.3389/fpls.2022.828482

**Published:** 2022-03-17

**Authors:** Yuanyuan Cui, Jianxin Bian, Yu Guan, Fangtao Xu, Xue Han, Xingwang Deng, Xiaoqin Liu

**Affiliations:** ^1^Institute of Advanced Agricultural Science, Peking University, Weifang, China; ^2^School of Advanced Agricultural Sciences, Peking University, Beijing, China

**Keywords:** peanut, ethylene signal, mechanical stress, EIN3, *AP2*/*ERF*, transcriptome

## Abstract

Peanut is an important oil and economic crop widely cultivated in the world. It has special characteristics such as blooming on the ground but bearing fruits underground. During the peg penetrating into the ground, it is subjected to mechanical stress from the soil at the same time. It has been proved that mechanical stress affects plant growth and development by regulating the ethylene signaling-related genes. In this study, we identified some genes related to ethylene signal of peanut, including 10 ethylene sensors, two constitutive triple responses (*CTR*s), four ethylene insensitive 2 (*EIN2*s), four ethylene insensitive 3 (*EIN3*s), six EIN3-binding F-box proteins (*EBF*s), and 188 Apetala2/ethylene-responsive factors (*AP2/ERF*s). One hundred and eighty-eight *AP2/ERF*s were further divided into four subfamilies, 123 *ERF*s, 56 *AP2*s, 6 Related to ABI3/VP1 (*RAV*s), and three *Soloist*s, of them one hundred and seventy *AP2/ERF* gene pairs were clustered into segmental duplication events in genome of *Arachis hypogaea*. A total of 134, 138, 97, and 150 *AhAP2/ERF* genes formed 210, 195, 166, and 525 orthologous gene pairs with *Arachis duranensis*, *Arachis ipaensis*, *Arabidopsis thaliana*, and *Glycine max*, respectively. Our transcriptome results showed that two *EIN3*s (*Arahy.J729H0* and *Arahy.S7XF8N*) and one *EBF*s (*Arahy.G4JMEM*) were highly expressed when mechanical stress increased. Among the 188 *AhAP2/ERF* genes, there were 31 genes with the fragments per kilobase of exon model per million mapped fragments (FPKM) ≥ 100 at least one of the 15 samples of Tifrunner. Among them, three *AhAP2/ERF*s (*Arahy.15RATX*, *Arahy.FAI7YU*, and *Arahy.452FBF*) were specifically expressed in seeds and five *AhAP2/ERF*s (*Arahy.HGAZ7D*, *Arahy.ZW7540*, *Arahy.4XS3FZ*, *Arahy.QGFJ76*, and *Arahy.AS0C7C*) were highly expressed in the tissues, which responded mechanical stress, suggesting that they might sense mechanical stress. Mechanical stress simulation experiment showed that three *AhAP2/ERF*s (*Arahy.QGFJ76*, *Arahy.AS0C7C*, and *Arahy.HGAZ7D*) were sensitive to mechanical stress changes and they all had the conservative repressor motif (DLNXXP) in the C-terminus, indicated that they might transmit mechanical stress signals through transcriptional inhibition. This study reveals the regulatory landscape of ethylene signal-related genes in peanut, providing valuable information for the mining of target genes for further study.

## Introduction

Cultivated peanut [*Arachis hypogaea* (*A. hypogaea*) L.], belonging to family Leguminosae, originated in South America (Bertioli et al., 2019). It is an important oil and economic crop, supplying oil and protein for human beings, which widely planted in tropical and subtropical areas. As the harvesting organ of peanut, pod is the key factor affecting peanut yield and quality (Liu et al., 2020). However, the molecular mechanism of peanut pod growth and development is still not clear; an in-depth understanding of this process has great significance for increasing peanut yield and improving peanut quality.

The peanut pod has unique characteristics such as it blooms on the ground but bears fruits underground. After pollination, the embryo temporarily stops developing and the ovary stalk continuously elongates, pushing the ovary toward the ground, called “gynophore” (Kumar et al., 2019). When the ovary entering into the soil, the environmental conditions change, including dark conditions, mechanical stress, moisture and nutrition, etc. The embryo continues to develop and eventually forms a pod. Previous studies have shown that mechanical stress is one of the important factors affecting the development of peanut pods (Ziv and Zamski, 1975; Zamski and Ziv, 1976; Kumar et al., 2019).

It has been proved that ethylene plays a remarkable role in growth and developmental changes in a process triggered by mechanical stress (Goeschl et al., 1966; Okamoto and Takahashi, 2019; Wu et al., 2020). Mechanical stress results in a rapid increase in the amount of ethylene and the activity of 1-aminocyclopropane-1-carboxylic acid (ACC) synthase, which is a key enzyme in ethylene synthesis (Eisinger, 1983; Biro and Jaffe, 1984; Emery et al., 1994). During the penetration, the amount of ethylene released from buried gynophore was twice as much as the aerial gynophore (Shlamovitz et al., 1995), indicating that ethylene is involved in the pod development.

Ethylene signal transduction begins with ethylene sensor family and ends with ethylene insensitive 3/ethylene insensitive 3-like (EIN3/EIL) and ethylene-responsive factor (ERF) family (Potuschak et al., 2003). Ethylene is perceived by five membrane-bound receptors: two ethylene receptors (ETR1 and ETR2), two ethylene response sensors (ERS1 and ERS2), and one ethylene insensitive 4 (EIN4). At low ethylene concentration, the receptors bind to constitutive triple response 1 (CTR1), which then phosphorylates ethylene insensitive 2 (EIN2). Due to the degradation of phosphorylated EIN2, the downward transmission of ethylene signal is inhibited. When the concentration of ethylene increases, ethylene molecules bind to receptors, inactivating CTR1 and unable to phosphorylate EIN2. At the same time, the N-terminal of the dephosphorylated EIN2 is cutoff and transported from the endoplasmic reticulum to the nucleus, stabilizing the transcription factor EIN3/EIL1 and resulting in an increased protein level of EIN3/EIL1 in nucleus. Furthermore, EIN3/EIL1 transmits ethylene signals by regulating the expression of ERFs and other ethylene-responsive genes (Guo and Ecker, 2004; Chen et al., 2005). EIN3-binding F-box protein (EBF) is located in the nucleus and interacts with EIN3/EIL; it regulated the ethylene signaling by modulating the stability of EIN3/EIL1 proteins. Overexpression of EBF1 results in plants insensitive to ethylene (Potuschak et al., 2003).

Apetala2 (AP2)/ERF superfamily is a large class of transcription factors in plants and shares a conserved AP2 domain (Perata, 2013). This superfamily is divided into three subfamilies: ERF, AP2, and RAV (Sakuma et al., 2002; Nakano et al., 2006). AP2/ERF transcription factors have been reported to be widely involved in the mechanical stimulation during the growth and development of plants (Nishiuchi et al., 2002; Feng et al., 2020).

What genes are involved in ethylene signaling pathway during the peanut pod development? According to the sequencing of tetraploid cultivated peanut (Bertioli et al., 2019; Chen et al., 2019; Zhuang et al., 2019), genes of ethylene signaling pathway, including the whole AP2/ERF superfamily in cultivated peanut, were identified in this study. Their gene structure, chromosome distribution, evolutionary relationships, and transcriptional expression during the pod development were analyzed. These results provide valuable information for clarifying regulatory mechanisms of mechanical stress in the process of peanut pod development.

## Materials and Methods

### Identification of Genes Involved in Ethylene Signaling Pathway in *Arachis hypogaea*

The genome data of *A. hypogaea* cv. Tifrunner, a runner-type peanut (registration number CV-93, PI 644011), were downloaded from https://www.peanutbase.org/home. The genomic data of *Arabidopsis* AP2/ERF sequences were downloaded from the arabidopsis information resource (TAIR).^1^ The ethylene pathway-related genes identified in *Arabidopsis thaliana* (*A. thaliana*) were used as clue to blast the peanut genome and these obtained genes were confirmed by the Conserved Domain Database (CDD).^2^ For *AP2/ERF* gene family, the Markov (HMM) file of AP2 domain (PF00847) downloaded from the Pfam database^3^ was used to retrieve *AP2/ERF* genes through hidden Markov marker and profiles (HMMER) version 3.0. The final family members of AP2/ERF were identified by a combination of basic local alignment search tool (BLAST) and HMM.

### Analysis of Phylogenetic, Gene Structure, Promoter, and Chromosomal Mapping

The longest transcripts of protein sequences of genes were selected for bioinformatics analysis and alternative splicing was obtained through genome annotations. MEGA and PlantCare^4^ were used to construct evolutionary trees [using maximum likelihood (ML)] and identify the putative cis-regulatory elements. The visualization of these information and chromosomal mapping were realized through TBtools (Chen et al., 2020).

### Gene Duplication Analysis

Genomic data of *Arachis duranensis*, *Arachis ipaensis*, and *Glycine max* can be downloaded from the following website https://www.peanutbase.org/home and https://v1.legumefederation.org/data/v2/Glycine/max. Interspecific and intraspecific syntenic analysis was performed by using Multiple Collinearity Scan toolkit (MCScanX) with the default parameters. The visualization is achieved through TBtools (Chen et al., 2020).

### Plant Material, Transcriptome, and Expression Analysis of Mechanical Stress Simulation Experiment

Haihua 1, a peanut cultivar, was planted at the Experimental Station of Institute of Advanced Agricultural Sciences of Peking University. The pod shells were the tissue that felt mechanical stress; thus, the pericarp of pods with a transverse diameter of about 15 mm (about 10 days after penetration into soil) was selected as experimental materials (Supplementary Figure 1A). The pods just dug out of the soil were the control group (CK, day 1). The pods out of the soil bagged with black breathable paper bag were the treatment group, simulating mechanical stress loss (Supplementary Figure 1B). Samples harvested after 10 (day 1), 34 (day 2), and 58 (day 3) h of treatment, termed as D1, D2, and D3, respectively. The pods were buried back into the soil and harvested after 48 h for simulating mechanical stress recovery, termed as recovery (RE) (day 5) (Supplementary Figure 1C). Each group had 15 pods, three biological replicates of each group. The pod shells of pods from the control group and the treatment group were isolated and stored at −80°C.

Ribonucleic acid was isolated from the thoroughly ground samples of pod shell with Takara MiniBEST Plant RNA Extraction Kit (Takara, Dalian, China). The complementary DNA (cDNA) libraries were constructed and then sequenced on the Illumina NovaSeq 6000. The original image data obtained were transformed into sequence data by consensus assessment of sequence and variation (CASAVA) base recognition. High-quality sequence data were obtained by removing reads containing adapter and low-quality sequences. Reference genome and gene model annotation files were downloaded from https://www.peanutbase.org/home and paired-end clean reads were aligned to the reference genome using Hisat2 (version 2.0.5). The RNA sequencing (RNA-Seq) data of this experiment had been deposited in the National Center for Biotechnology Information (NCBI) [sequence read archive (SRA) accession: PRJNA770556]. Besides for our own transcriptome data, gene expression analysis also referenced the previous transcriptome publications, a developmental transcriptome of Tifrunner (Clevenger et al., 2016).

### Quantitative PCR Validation

The cDNA was synthesized with the PrimeScript RT Reagent Kit (Takara, Dalian, China). The gene primers were designed by Primer3^5^ and the *elongation factor 1B* (*Arahy.E3HYWR*) was used for normalization (Supplementary Table 1). The qPCR was performed on the ABI 7500 Fast using the TB Green^®^ Premix Ex Taq™ (Takara, Dalian, China), with three replicates. The reaction conditions were: 95°C for 5 min, 40 thermal cycles of 95°C for 30 s, and then 60°C for 30 s. The 2^–^ΔΔ^CT^ method was used for relative quantification analysis (Livak and Schmittgen, 2001).

### Prediction of Target Genes and Interacted Proteins

Prediction of target genes and interaction network were referenced to homologous genes in *A. thaliana* according to http://planttfdb.gao-lab.org/index.php and https://cn.string-db.org, respectively (Tian et al., 2019; Szklarczyk et al., 2021). Only interactions that had been demonstrated by experimental evidence were shown.

## Results

### Identification and Expression Analysis of Ethylene Sensors, *Constitutive Triple Responses*, *Ethylene Insensitive 2*, *Ethylene Insensitive 3/Ethylene Insensitive 3-Like*, and *Binding F-Box Proteins*

**FIGURE 1:**
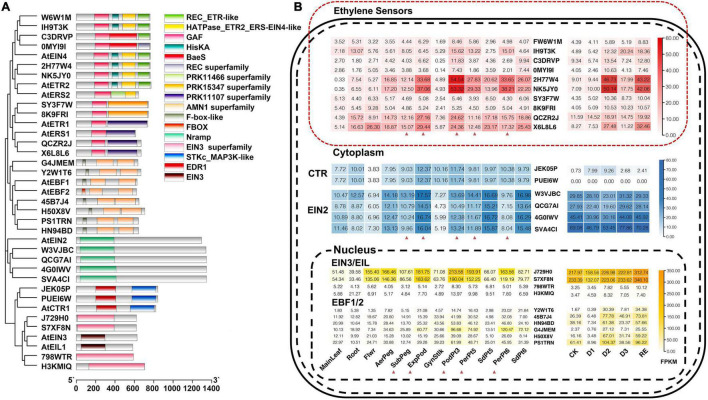
Conserved domain and expression of ethylene sensors, constitutive triple responses (*CTR*s), ethylene insensitive 2 (*EIN2*s), ethylene insensitive 3/ethylene insensitive 3-like (*EIN3/EIL*s), and EIN3-binding F-box proteins (*EBF*s). **(A)** Conserved domain of ethylene sensors, *CTR*s, *EIN2*s, *EIN3/EIL*s, and *EBF*s in *Arachis hypogaea* (*A. hypogaea*) genome; **(B)** Developmental expression analysis of ethylene sensors, *CTR*s, *EIN2*s, *EIN3/EIL*s, and *EBF*s in *A. hypogaea* genome. The red triangle indicates the tissues that experience mechanical stress.

According to homologous genes in *Arabidopsis*, ten ethylene sensors, two *CTR*s, four *EIN2*s, four *EIN3/EIL*s, and six *EBF*s were identified in *A. hypogaea* cv. Tifrunner (Figure 1A and Supplementary Table 2). They were located on the corresponding chromosomes of the A and B subgenomes, each A gene corresponded to a B gene (Supplementary Figure 2). Expression analysis showed that the expression of two ethylene sensors (*Arahy.2H77W4* and *Arahy.NK5JY0*) in pod was significantly higher than that in other tissues and increased after penetration and expansion. Mechanical stress simulation experiment showed that they increased after 34 h of losing pressure and then decreased. When the pressure was restored, their expression rose again (Figure 1B). This result indicated that *Arahy.2H77W4* and *Arahy.NK5JY0* might be the main ethylene sensors functioning in the pod development. Besides, the expression of two *AhEIN3/EIL*s (*Arahy.J729H0* and *Arahy.S7XF8N*) in reproductive organ, especially in pericarps, was significantly higher than leaf and root. Because pericarp first sensed the changes from the peripheral environment, they suggesting the significance of *Arahy.J729H0* and *Arahy.S7XF8N* in the pod development (Figure 1B). In addition, an *AhEBF*s (*Arahy.G4JMEM*) also showed similar expression pattern with *AhEIN3/EIL*s.

Interestingly, in our mechanical stress simulation experiment using cultivar Haihua 1, all the six *AhEBF*s decreased after 10 h of mechanical stress loss, then significantly increased on the second day of mechanical stress loss, then slightly decreased on the third day, and increased again when mechanical stress was restored 2 days later (Figure 1B). They response to the changes of the external environment and there was no positive or negative correlation between their expression patterns and mechanical stress, so it was speculated that *AhEBF*s might be act as signal transducers, sending the external signals to downstream genes. If the external environment did not change, their expression would not change dramatically.

### Evolution and Alternative Splicing Analysis *of Apetala2*/*Ethylene- Responsive Factor* Transcription Factors in *Arachis hypogaea*

**FIGURE 2:**
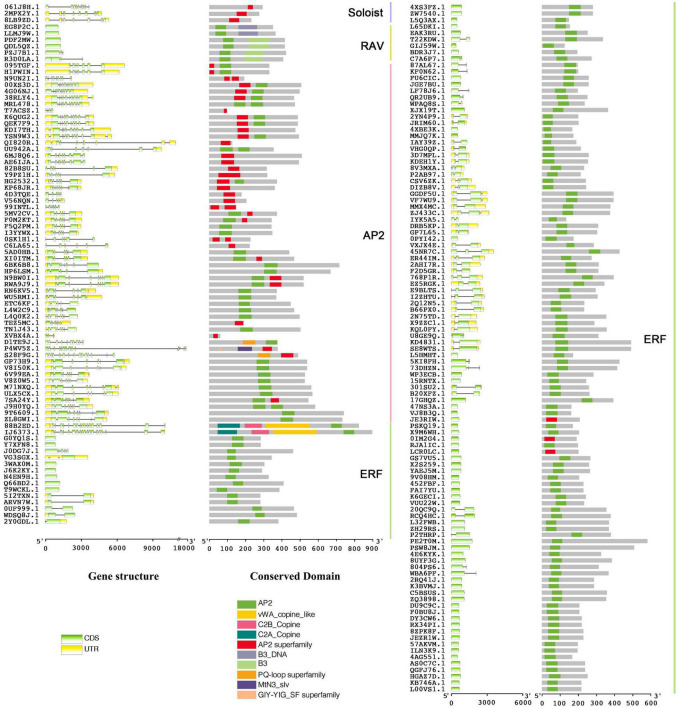
Gene structure and conserved domain of 188 Apetala2/ethylene-responsive factors (*AhAP2/ERF*s) in *A. hypogaea* genome.

**FIGURE 3:**
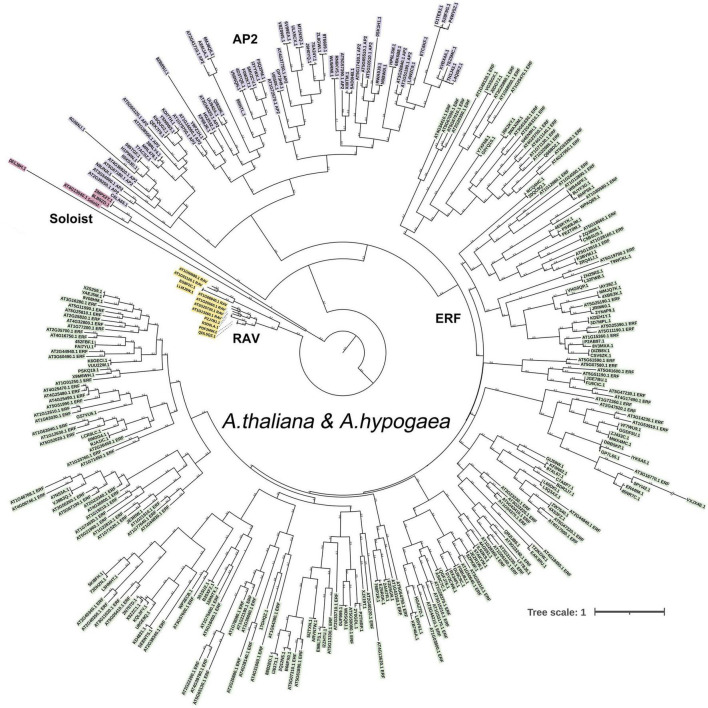
Phylogenetic tree of AhAP2/ERFs and AtAP2/ERFs.

Combining BLAST and HMM results, there were 188 *AP2/ERF* genes identified in *A. hypogaea* cv. Tifrunner. The CDD and phylogenetic analysis showed that 123 genes belonged to ERF subfamily, 56 genes belonged to AP2 subfamily, six genes belonged to RAV subfamily, and three genes belonged to Soloist. Among AP2 subfamily, 21 genes contained two AP2 domains and 35 genes contained one AP2 domain (Figures 2, 3). Gene structure showed that most of AP2 subfamily members and three Soloists had numerous introns. However, ERF subfamily members contained only 2 or less introns, except *Arahy.88B2ED* and *Arahy.IJ6373* (Figure 2).

**FIGURE 4:**
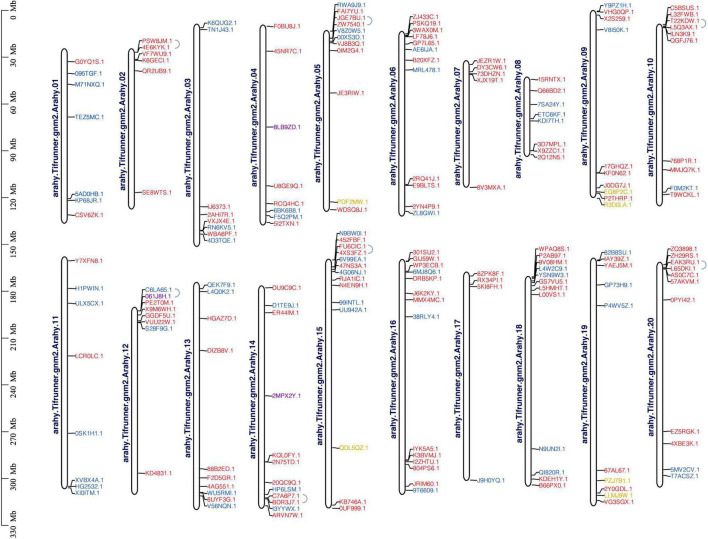
Chromosomal distribution and tandem duplication of *AhAP2/ERF*s in *A. hypogaea* genome. Red gene ID: ERFs; blue gene ID: AP2; yellow gene ID: RAV; purple gene ID: Soloist.

One hundred and eighty-eight *AP2/ERF* genes were unevenly distributed on 20 chromosomes, but they did not form a correspondence between A and B subgenome (Figure 4 and Supplementary Figure 3). The possible causes were the loss of gene annotation or the increase of members after the formation of tetraploid. The maximum number of *AP2/ERF*s were located on chromosomes 15 and 16, both with 14 *AP2/ERF*s. Chromosome 17 had the minimum number of *AP2/ERF*, only 4. Three Soloists were distributed on chromosomes 4, 12, and 14, respectively. The six RAV family members were distributed on chromosomes 5, 9, 15, and 19. Chromosomes 2 and 7 only had ERF family members (Figure 4 and Supplementary Figure 3). Fourteen *AhAP2/ERF* genes were clustered into seven pairs of tandem duplication event on chromosomes 2, 5, 10, 12, 14, 15, and 20. The correspondence between 2, 5, 10 and 12, 15, 20 suggests that tandem repetition preceded the formation of the tetraploid (Figure 4). One hundred and seventy *AP2/ERF* gene pairs were clustered into segmental duplication events in genome of *A. hypogaea* cv. Tifrunner, suggesting the high conservation of the *AP2/ERF* gene family, which played crucial roles in the expansion of *AP2/ERF* gene family (Supplementary Figure 3 and Supplementary Table 3).

**FIGURE 5:**
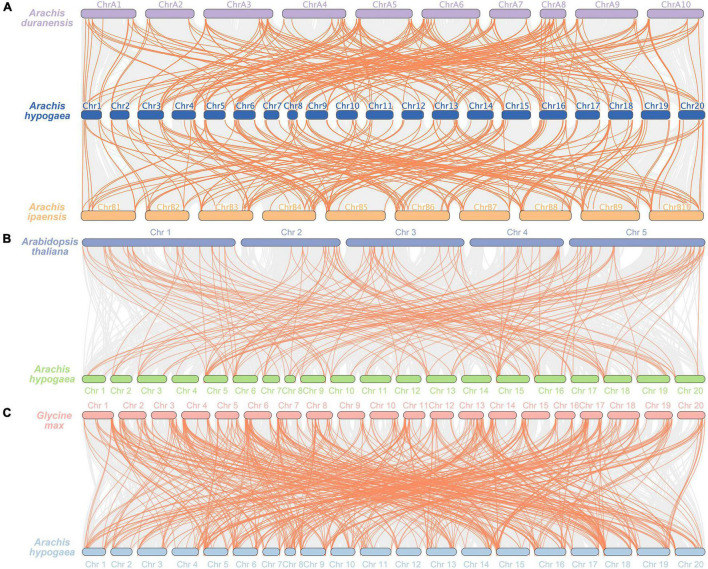
Synteny analysis of *AP2/ERF* genes between *A. hypogaea* and four other plant species. Gray lines indicated the syntenic gene pairs within genome, while tangerine lines highlighted the syntenic *AP2/ERF* gene pairs. **(A)** Synteny analysis of *AP2/ERF* genes between *A. hypogaea*, *Arachis duranensis*, and *Arachis ipaensis*; **(B)** Synteny analysis of *AP2/ERF* genes in *A. hypogaea* vs. *Arabidopsis thaliana* (*A. thaliana*); **(C)** Synteny analysis of *AP2/ERF* genes in *A. hypogaea* vs. *Glycine max*.

In order to further study the phylogenetic and evolutionary relationships of *AP2/ERF* gene family in *A. hypogaea*, three comparative syntenic maps associated with *Arachis duranensis*, *Arachis ipaensis*, *A. thaliana*, and *Glycine max* were performed (Figure 5). A total of 134 and 138 *AhAP2/ERF* genes formed 210 and 195 orthologous gene pairs with 71 and 77 *AP2/ERF* genes in *Arachis duranensis* and *Arachis ipaensis*, respectively. *AhAP2/ERF* genes that were not found orthologous gene pairs with *Arachis duranensis* or *Arachis ipaensis* may indicate that these genes appeared after the heterozygote was formed (Figure 5A and Supplementary Table 4). A total of 97 and 150 *AhAP2/ERF* genes formed 166 and 525 orthologous gene pairs with 75 and 206 *AP2/ERF* genes in *A. thaliana* and *Glycine max*, respectively (Figures 5B,C and Supplementary Table 4). Apparently, soybeans had a closer relatedness with peanuts. Some *AhAP2/ERF* genes formed two or more orthologous gene pairs with all the four species, such as *Arahy.00XS3D* and *Arahy.095TGF*, suggesting the conservation in evolution and the importance of gene function (Supplementary Table 4).

According to the genome annotations, we found that a total of 12 *AhAP2/ERF* genes had alternative splices (ASs), including exon skip (ES), alternate promoter (AP), alternate terminator (AT), and retained intron (RI). AP or AT was found to exist in 6 *AhAP2/ERFs*, which was the main splicing method; RI was found to exist in 5 *AhAP2/ERFs* and ES was found to exist in 2 *AhAP2/ERFs* (Supplementary Figure 4).

### Developmental Transcriptome Changes of *Apetala2*/*Ethylene-Responsive Factor* Genes

**FIGURE 6:**
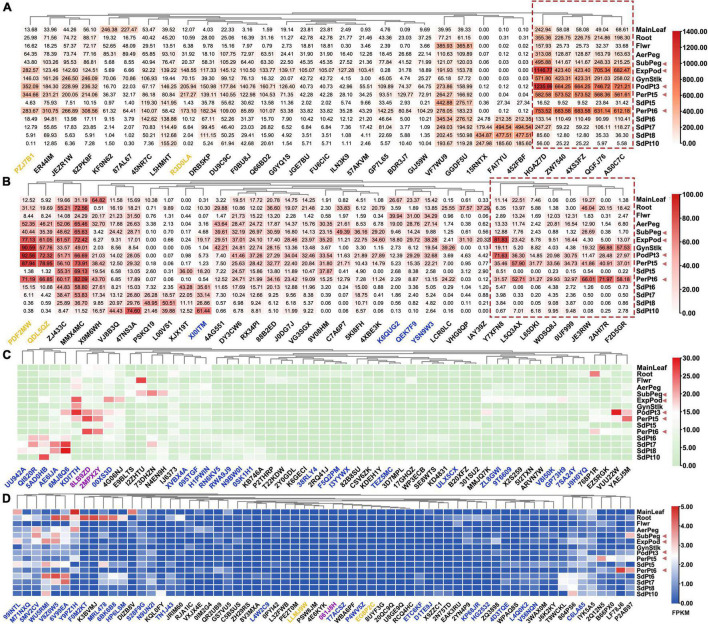
Gene express patterns of 188 *FcAP2/ERF* genes in 15 samples in *A. hypogaea* cv. Tifrunner. Main leaf, main stem leaf; Root, roots of 10 days postemergence; Flwr, petals, keel, and hypanthium sepals; AerPeg, elongating aerial pegs; Subpeg, elongating subterranean pegs; GynStlk, Pattee 1 stalk of gynophore; PodPt3, Pattee 3 pod; PerPt5, Pattee 5 pericarp; SdPt5, Pattee 5 seed; PerPt6, Pattee 6 pericarp; SdPt6, Pattee 6 seed; SdPt7, Pattee 7 seed; SdPt8, Pattee 8 seed; SdPt10, Pattee 10 seed (Clevenger et al., 2016). Black gene ID: ERFs; blue gene ID: AP2; yellow gene ID: RAV; purple gene ID: Soloist. They were divided into the four groups, red dotted boxes indicated *FcAP2/ERF*s that might be involved in mechanical pressure signal transduction. The red triangle indicates the tissues that experience mechanical stress. **(A)** The maximum FPKM value was greater than 100; **(B)** The maximum FPKM value was between 30 and 100; **(C)** The maximum FPKM value was between 5 and 30; **(D)** The maximum FPKM value was less than 5.

Referenced to developmental transcriptome map of Tifrunner from previous transcriptome publications (Clevenger et al., 2016), 188 *AhAP2/ERF* genes were divided into four categories according to their highest FPKM: (a) 31 *AhAP2/ERF*s, FPKM ≥ 100 in at least one of the 15 samples; (b) 35 *AhAP2/ERF*s, 30 ≤ FPKM ≤ 100 in at least one of the 15 samples; (c) 57 *AhAP2/ERF*s, 5 ≤ FPKM ≤ 30 in at least one of the 15 samples; and (d) 65 *AhAP2/ERF*s, 0 ≤ FPKM ≤ 5 in all the 15 samples (Figure 6 and Supplementary Table 5). The high expression genes (categories a and b) were mainly ERF members, accounting for 80% of the number of highly expressed genes. Fifty-two of 56 *AP2* genes and all the Soloists were in the low expression categories (categories c and d). There were two RAVs and 29 ERFs in “a” category and three *AhAP2/ERF*s (*Arahy.15RATX*, *Arahy.FAI7YU*, and *Arahy.452FBF*) were specifically expressed in seeds and highly expressed in late seed maturation. Five *AhAP2/ERF*s (*Arahy.HGAZ7D*, *Arahy.ZW7540*, *Arahy.4XS3FZ*, *Arahy.QGFJ76*, and *Arahy.AS0C7C*) were highly expressed in Expod, PodPt3, PerPt5, and PerPt6, which were the tissues that sensed mechanical stress, suggesting that they might be related to mechanical stress sensing (Figure 6A). Similar expression patterns were found in 8 *AhAP2/ERF*s from “b” category. Interestingly, their expression in seeds were relatively lower than other tissues (Figure 6B), indicating that they might be related to response to external changes.

### Promoter Analysis of Highly Expressed *Apetala2*/*Ethylene-Responsive Factor* Genes

**FIGURE 7:**
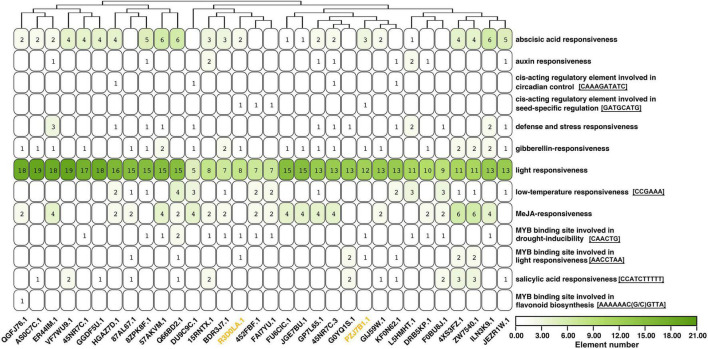
Promoter analysis of 31 highly expressed *AhAP2/ERF* genes. Black gene ID: ERFs; yellow gene ID: RAV. Only elements with the same sequence were displayed. The number represents the number of conserved domains and those with zero number is not marked.

The 2,000 bp upstream sequences of 31 *AhAP2/ERF*s (FPKM ≥ 100 in at least one of the 15 samples) were extracted for promoter analysis. The most abundant elements were light responsiveness, followed by abscisic acid (ABA) responsiveness and methyl jasmonate (MeJA) responsiveness, suggesting that their expression was mainly regulated by light, ABA, and MeJA (Figure 7 and Supplementary Table 6). Four *AhAP2/ERF*s (*Arahy.HGAZ7D*, *Arahy.DU9C9C*, *Arahy.45NR7C.3*, and *Arahy.KF0N62*) contained a circadian control element (CAAAGATATC motif). Among them, *Arahy.HGAZ7D* and *Arahy.DU9C9C* were highly expressed in the tissues that sensed mechanical stress, especially *Arahy.HGAZ7D* (Figure 6A), indicating that circadian rhythms were involved in the pod development. There were 13 *AhAP2/ERF*s contained at least one salicylic acid-responsiveness element (CCATCTTTTT motif). *Arahy.4XS3FZ* and its homolog (*Arahy.ZW7540*) had the most number (3) and their protein sequence showed 43.4 and 41.9% similarity to NtERF1 and AtERF2, which were involved in disease resistance pathways, suggesting *Arahy.4XS3FZ* and *Arahy.ZW7540* might be related to disease resistance. In addition, there were four *AhAP2/ERF* (*Arahy.R3D0LA*, *Arahy.452FBF*, *Arahy.FAI7YU*, and *Arahy.PZJ7B1*) that contained a cis-acting regulatory element involved in seed-specific regulation (GATGCATG motif). Significantly, *Arahy.R3D0LA* and *Arahy.PZJ7B1* were slowly expressed in seeds, while *Arahy.452FBF* and *Arahy.FAI7YU* were specifically expressed in the seed (Figures 6A, 7).

### Expression Changes of *Apetala2*/*Ethylene-Responsive Factor* Genes in Response to Mechanical Stress

**FIGURE 8:**
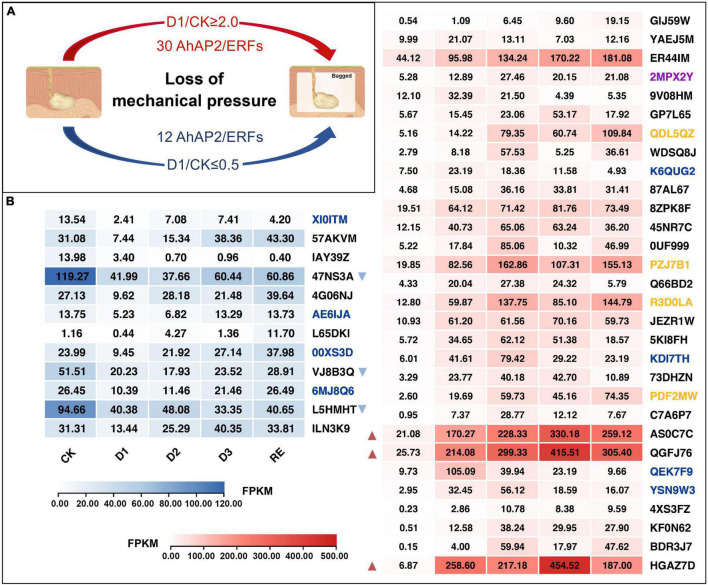
Expression changes of *AhAP2/ERF* genes in mechanical stress simulation experiment. Black gene ID: ERFs; blue gene ID: AP2; yellow gene ID: RAV; purple gene ID: Soloist. CK: control group, pods just out of the soil; D1: treatment group, 10 h bagging; D2: 34 h bagging; D3: 58 h bagging; RE: pods buried back into the soil for 48 h (Supplementary Figure 1C: Flowchart). **(A)** Number of differentially expressed *AhAP2/ERF* genes after 10 h loss of mechanical stress; **(B)** The FPKM value of *AhAP2/ERF* genes. The red and blue triangles indicated genes with high upregulation or downregulation, respectively.

Mechanical stress simulation experiment showed that 30 *AhAP2/ERF*s were upregulated 2-fold change and 12 *AhAP2/ERF*s were downregulated 2-fold change after out of the soil (losing the mechanical stress) (Figure 8A). In the downregulated group, there were three *AhAP2/ERF*s (*Arahy.47NS3A*, *Arahy.VJ8B3Q*, and *Arahy.L5HMHT*) with higher expression levels and downregulated by 0.35, 0.39, and 0.43 times in D1 vs. CK, respectively. In the upregulated group, there were also three *AhAP2/ERF*s (*Arahy.QGFJ76*, *Arahy.AS0C7C*, and *Arahy.HGAZ7D*) with higher expression levels and upregulated by 8.08, 8.32, and 37.63 times in D1 vs. CK, respectively (Figure 8B). Coincidentally, these three unregulated *AhAP2/ERF*s were included in the five *AhAP2/ERF*s that were highly expressed in Expod, PodPt3, PerPt5, and PerPt6. Four RAV members with higher expression levels were upregulated by 2.76–7.57 times in D1 vs. CK (Figures 6A, 8B).

### Validate the Transcriptome of Mechanical Stress Simulation by Real-Time Quantitative PCR

**FIGURE 9:**
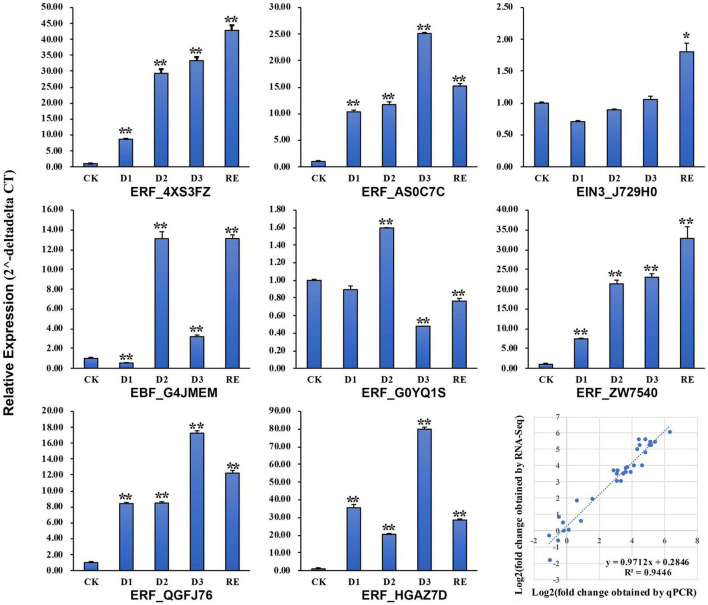
Validation of the RNA sequencing (RNA-Seq) results by real-time quantitative PCR (qRT-PCR). *, **Significantly different at *p* ≤ 0.05 and 0.01, respectively.

Eight genes including 1 *EIN3*, 1 *EBF*, and 6 *AP2/ERF*s were selected to verify the transcriptome results by qRT-PCR. The expression patterns of those genes were consistent with the transcriptome results (Figure 9) and linear regression analysis revealed a high correlation coefficient (*R*^2^ = 0.94; Figure 9).

### Protein Sequence Alignment, Target Genes, and Protein Interaction Networks

**FIGURE 10:**
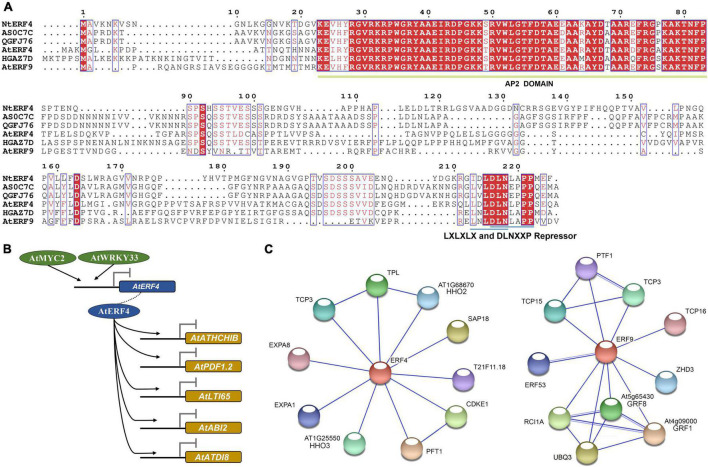
Protein sequence alignment, target genes, and interaction network analysis of key AP2/ERFs according to *A. thaliana* homologs. **(A)** Protein sequence alignment of AtERF4, AtERF9, NtERF4, Arahy.AS0C7C, Arahy.QGFJ76, and Arahy.HGAZ7D; **(B)** Target genes of *Arabidopsis* homologs; **(C)** Interaction network relationships of *Arabidopsis* homologs.

To explore the possible genetic functions of key three *AhAP2/ERF*s, their protein sequences were further studied. The protein sequences of *Arahy.QGFJ76* and *Arahy.AS0C7C* showed only one amino acid difference and they were homologous genes located on chromosomes 10 (subgenome A) and 20 (subgenome B), respectively. The protein sequence similarity between *Arahy.HGAZ7D* and *Arahy.QGFJ76* or *Arahy.AS0C7C* was 75% and they contained one AP2 domain, belonging to ERF subfamily (Figure 10A). Furthermore, the protein sequences of *Arahy.QGFJ76* and *Arahy.AS0C7C* showed 46.6 and 46.2% similarity to NtERF4 and the protein sequences of *Arahy.HGAZ7D* showed 44.7, 46.0, and 47.3% similarity to NtERF4, AtERF4, and AtERF9, respectively. NtERF4, AtERF4, and AtERF9 were all the transcriptional repressors. Coincidentally, repressor motifs (LXLXLX and DLNXXP) were also found in the C-terminus of these three *AhAP2/ERF*s (Figure 10A).

In *Arabidopsis*, the homologous gene AtERF4 of *Arahy.HGAZ7D* could bind to the promoters of *AtATHCHIB*, *AtPDF1.2*, *AtLTI65*, *AtABI2*, and *AtATDI8* to response to hormonal signals such as ethylene, jasmonic acid, and ABA and inhibit their expressions. Furthermore, the expression of AtERF4 was inhibited by *Arabidopsis thaliana* myelocytomatosis 2 (AtMYC2) and *Arabidopsis thaliana* WRKY (WRKY amino acid sequence at the N-terminus) 33 (AtWRKY33) (Figure 10B). Besides, interaction network analysis showed that AtERF4 could interacted with 10 proteins and might form two protein complexes, one with AtTCP3, *Arabidopsis thaliana* TOPLESS (AtTPL), and *Arabidopsis thaliana* hypersensitivity to low phosphate-elicited primary root shortening 1 homolog2 (AtHHO2) and the other with *Arabidopsis thaliana* cyclin-dependent kinase E-1 (AtCDKE1) and *Arabidopsis thaliana* photochrome and flowering time 1 (AtPFT1) (Figure 10B). Although, the target gene of AtERF9 had not been elucidated, AtERF9 could also interacted with 10 proteins and might form two protein complexes, one with AtTCP3, AtTCP15, and *Arabidopsis thaliana* plastid transcription factor 1 (AtPTF1)/TCP13 and the other with *Arabidopsis thaliana* growth-regulating factor 1 (AtGRF1), AtGRF8, *Arabidopsis thaliana* rare cold inducible 1A (AtRCI1A), and Polyubiquitin 3 (UBQ3) (Figure 10C). Both the AtERF4 and AtERF9 interacted with T-complex protein (TCPs). TCP transcription factors play an important role in plant organ morphogenesis by regulating the expression of boundary-specific genes (Koyama et al., 2007). Therefore, it was speculated that *AhERFs* might regulate the morphological change from gynophore to pod through TCPs.

## Discussion

The ethylene signal-related genes in peanut were systematically analyzed in this study. Ethylene signal-related genes including *AP2/ERFs* in cultivated peanut were identified. Combined developmental- and mechanical stress-related transcriptome provides a comprehensive understanding of these gene changes related to peg penetration and pod development. Two *AhEIN3*s, one *AhEBF*s, and three *AhAP2/ERFs* may be the key genes for their high expression and expression differences.

This study revealed transcriptional changes of ethylene signal genes in peanut. However, the final executor of life activities is protein and there is a large amount of posttranscriptional regulation between transcription and protein (Floris et al., 2009). Therefore, transcription levels and protein levels are often inconsistent (Cui et al., 2019). Besides, posttranslational protein modifications including phosphorylation, ubiquitination, sumoylation, etc., are the core of many cellular signaling events (Dai Vu et al., 2018), they are greatly increasing the complexity of biological events. Currently, there are few studies on posttranscriptional protein modification of EIN3 and AP2/ERFs. However, advances in mass spectrometry have made it possible to discover more posttranscriptional modifications. Therefore, the posttranslational protein modifications will be necessary for further study of key genes.

Ethylene signal-related genes including *AP2/ERFs* in cultivated peanut were all located at the two ends of chromosomes (Supplementary Figure 2 and Figure 4), which are consistent with the previous description in the cultivated peanut genome; nonautonomous long terminal repeat (LTR) retrotransposons and Ty3-gypsy elements are mainly distributed in the middle of chromosomes, while genes are mainly distributed in the end (Bertioli et al., 2019; Chen et al., 2019). Comparative genomic analysis of wheat and rice showed that gene evolution occurred preferentially at the end of chromosomes and was associated with replication and differentiation associated with high recombination rates (See et al., 2006). Other comparative studies also showed that the region around telomeres was unstable and a hot spot in chromosome evolution, containing extensive rearrangement and segmental gene duplication (Eichler and Sankoff, 2003; Kellis et al., 2003). Therefore, the genes of cultivated peanut, such as those of other crops, are mainly distributed at both the ends of the chromosome, which are conducive to their own better evolution and expansion.

Our results showed that there were differences in the gene structure of different subfamilies of *AhAP2/ERFs* (Figure 2). It is particularly interesting that AP2 subfamily members contained more introns than other subfamilies. This also occurs in other plant such as pear (Li et al., 2018), tartary buckwheat (Liu et al., 2019), longan (Xu et al., 2020), sugarcane (Li et al., 2020), poppy (Yamada et al., 2020), creat (Yao et al., 2020), and pineapple (Zhang et al., 2021). There are two hypotheses about the origin of introns. One is that the intron is already present when the gene appears and the other is that the intron is inserted through transposition during evolution (Rodríguez-Trelles et al., 2006). Although the origin is still unknown, most early eukaryotic genes are rich in intron. Therefore, from an evolutionary perspective, intron poverty is later than intron abundance (Liu et al., 2021).

A single gene can expand the potential informational content of genomes, which plays a major role in the generation of proteomic and functional diversity in organisms (Blencowe, 2006). Introns play an important role in AS, which is ubiquitous under stress conditions (Chaudhary et al., 2019), as previously reported. Intron retention is a major phenomenon in AS in *Arabidopsis* and transcripts that retain introns are mainly related to stress and external/internal stimuli (Ner-Gaon et al., 2004). Nevertheless, the contribution of AS to proteome complexity is still elusive in plants. Why introns preferred AP2 subfamily in AP2/ERF family? It is speculated that AP2 subfamily is a reserved family of AP2/ERF and evolutionarily earlier than ERF subfamily. AP2 subfamily maintains variability in response to external changes, while ERF has evolved to maintain basal growth and development. The highly expressed genes were mainly members of ERF family, which seemed to support this hypothesis (Figure 6A). More details need further research to reveal.

Most of published data have been demonstrated that ethylene is involved in the plant growth and development (Bleecker and Kende, 2000; Dubois et al., 2018). Moreover, there is ample evidence that different stimulus, such as wounding, mechanical stress, waterlogging, and submergence, induce the burst of ethylene (Khan et al., 2020; Zhao et al., 2020). Mechanical stress has proved to play an important role in shaping architecture of plants (Hamant and Traas, 2010; Landrein and Ingram, 2019; Okamoto et al., 2021). Ethylene is the first plant growth regulator to be implicated in the response to mechanical stress (Goeschl et al., 1966). More recently, it has been reported that before the seedlings are unearthed, the pressure from the soil stimulates ethylene production and represses polygalacturonase involved in expansion 3 (PGX3) to facilitate seedling emergence from the soil in a dose-dependent manner through the transcription factor EIN3 (Wu et al., 2020). When peg penetrates into the soil, the mechanical stress increases and the light decreases. Our results showed that the expression of EIN3 had been at a high level (Figure 1B). EIN3 primarily binds to ethylene-related elements present in the promoter of AP2/ERFs to activate their transcription (Solano et al., 1998). In peanut, whether it regulates some AP2/ERFs or other genes in a dose-dependent manner? How this process is cross-regulated with light and other hormones? It requires further research.

Piezo is the most well-characterized mechanosensors in mammals. Upon activation, the Piezo channel facilitates the entry of calcium ion (Ca^2+^) inside the cell, translating the cellular signals (Coste et al., 2012). The sequences of Piezo family proteins are highly conserved in plants, in both the moss (*Physcomitrium patens*) and the small flowering plant *Arabidopsis*; the mutations of plant Piezo sensors change vacuolar morphology and growth patterns of apical growth cells (Radin et al., 2021). Moreover, AtPiezo responds to mechanical stimuli at the transcriptional level, playing a role in the perception of mechanical force in plant root cap and the flow of Ca^2+^ is involved in this process (Fang et al., 2021a,b; Mousavi et al., 2021). In peanut, four AhPiezo were identified, but they did not show any significant changes in transcription levels during mechanical stress changes (Supplementary Figure 5). Whether Piezo was involved in the formation of peanut pods and whether Piezo was associated with other hormonal signals are an interesting question in plants, waiting for our further exploration.

## Conclusion

In this study, the ethylene signal-related genes in peanut (*A. hypogaea* L.) were analyzed, including conserved domains, gene structure, evolutions, AS, cis-elements, and expression patterns. Ten ethylene sensors, two *CTR*s, four *EIN2*s, four *EIN3*s, six *EBF*s, and 188 *AP2/ERF*s were identified and their expression pattern was revealed according to published transcriptome data of Tifrunner and our own mechanical stress simulation transcriptome. The change of mechanical stress affected the expression of genes related to ethylene signaling pathway, in particular two *AhEIN3*s, one *AhEBF*s, and three *AhAP2/ERFs* were mostly affected, suggesting that they might play an important role in the pod development of peanut.

## Data Availability Statement

The datasets presented in this study can be found in online repositories. The names of the repository/repositories and accession number(s) can be found below: https://www.ncbi.nlm.nih.gov/, PRJNA770556.

## Author Contributions

XL and YC conceived this study. XL and XD supervised the research. YC performed the experiments. YC, XL, JB, and XH analyzed the data. XL, YC, XD, JB, YG, and FX prepared the manuscript. All the authors have read and approved the manuscript for publication.

## Conflict of Interest

The authors declare that the research was conducted in the absence of any commercial or financial relationships that could be construed as a potential conflict of interest.

## Publisher’s Note

All claims expressed in this article are solely those of the authors and do not necessarily represent those of their affiliated organizations, or those of the publisher, the editors and the reviewers. Any product that may be evaluated in this article, or claim that may be made by its manufacturer, is not guaranteed or endorsed by the publisher.

## Footnotes

1
https://www.arabidopsis.org

2
https://www.ncbi.nlm.nih.gov/cdd

3
http://pfam.xfam.org/

4
http://bioinformatics.psb.ugent.be/webtools/plantcare/html/

5
https://bioinfo.ut.ee/primer3/

## Supplementary Material

Supplementary Figure 1 Experimental materials and illustrations of mechanical pressure simulation experiment.

Supplementary Figure 2 Chromosomal distribution of ethylene sensors, constitutive triple responses (*CTR*s), ethylene insensitive 2 (*EIN2*s), ethylene insensitive 3/ethylene insensitive 3-like (*EIN3/EIL*s), and EIN3-binding F-box proteins (*EBF*s) in *Arachis hypogaea* (*A. hypogaea*) genome.

Supplementary Figure 3 Gene location and intragenomic collinearity of AhAP2/ERFs. Gray lines indicated all the collinearity in *A. hypogaea* genome, while colored lines highlighted the collinearity of AhAP2/ERFs.

Supplementary Figure 4 Alternative splice (AS) analysis of Apetala2/ethylene-responsive factor (*AP2/ERF*) genes in *A. hypogaea*. ES, exon skip; AP, alternate promoter; AT, alternate terminator; RI, retained intron. The red inverted triangle indicated the difference of AS.

Supplementary Figure 5 Protein sequence alignment and express pattern of *AhPiezo.*

Supplementary Table 1 The primers of quantitative PCR (qPCR).

Supplementary Table 2 The FPKM values of ethylene signal genes during developmental stage and in response to mechanical stress.

Supplementary Table 3 Segmental duplication Apetala2/ethylene-responsive factor (*AhAP2/ERF*) gene pairs in *Arachis hypogaea* (*A. hypogaea*) genome.

Supplementary Table 4 Segmental duplication *AhAP2/ERF* gene pairs in *A. hypogaea* and four other plant species.

Supplementary Table 5 The FPKM values of *AhAP2/ERFs* during developmental stage.

Supplementary Table 6 Promoter elements information.

Supplementary Table 7 The FPKM values of *AhAP2/ERFs* in response to mechanical stress.
