# Supplementary figures and images for: Genome-Wide Analysis and Expression Profiles of Ethylene Signal Genes and Apetala2/Ethylene-Responsive Factors in Peanut (Arachis hypogaea L.)
Source: Front Plant Sci. 2022 Mar 17;13:828482. doi: 10.3389/fpls.2022.828482 (PMC8968948; doi:10.3389/fpls.2022.828482)

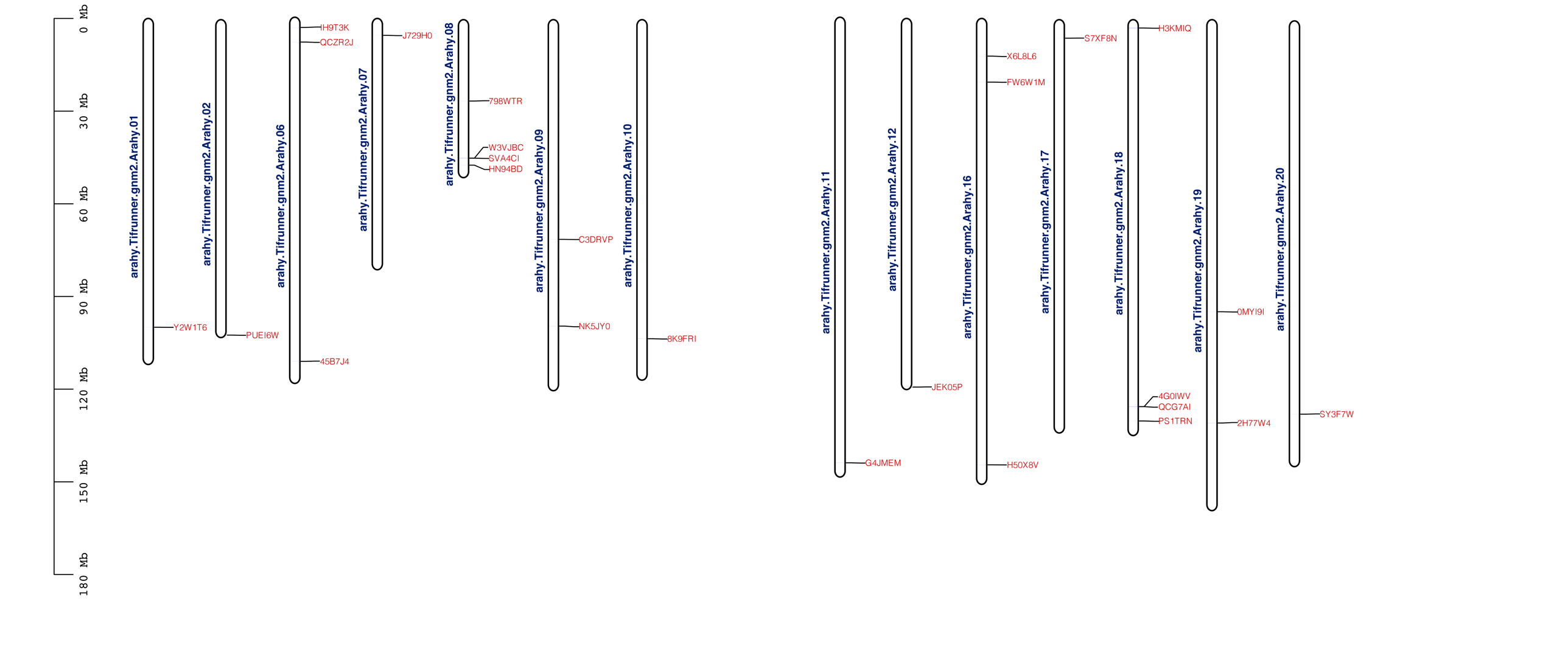

Supplement: Supplementary Figure 2 — Chromosomal distribution of ethylene sensors, constitutive triple responses (CTRs), ethylene insensitive 2 (EIN2s), ethylene insensitive 3/ethylene insensitive 3-like (EIN3/EILs), and EIN3-binding F-box proteins (EBFs) in Arachis hypogaea (A. hypogaea) genome. [file Image_2.JPEG]

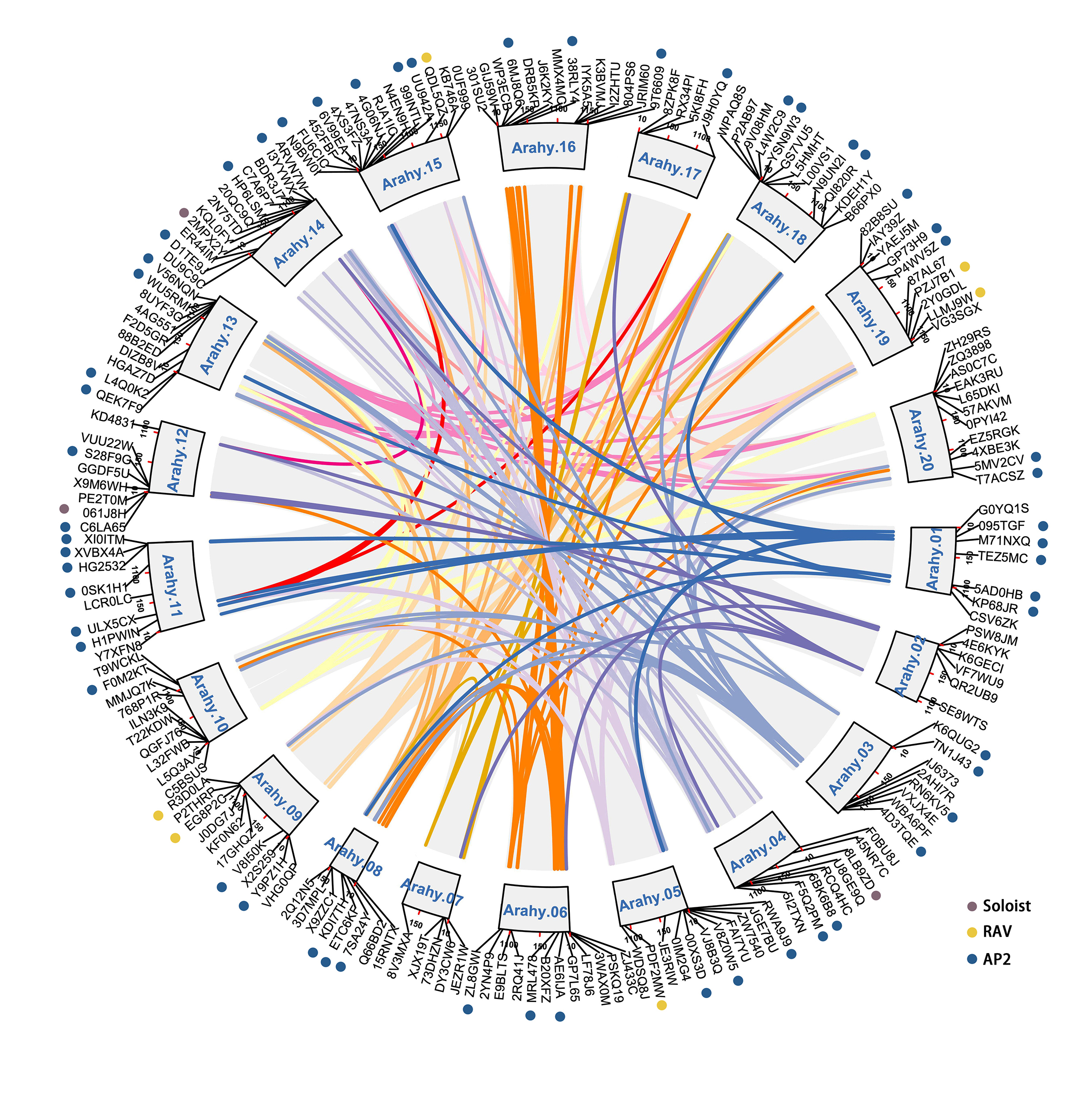

Supplement: Supplementary Figure 3 — Gene location and intragenomic collinearity of AhAP2/ERFs. Gray lines indicated all the collinearity in A. hypogaea genome, while colored lines highlighted the collinearity of AhAP2/ERFs. [file Image_3.JPEG]

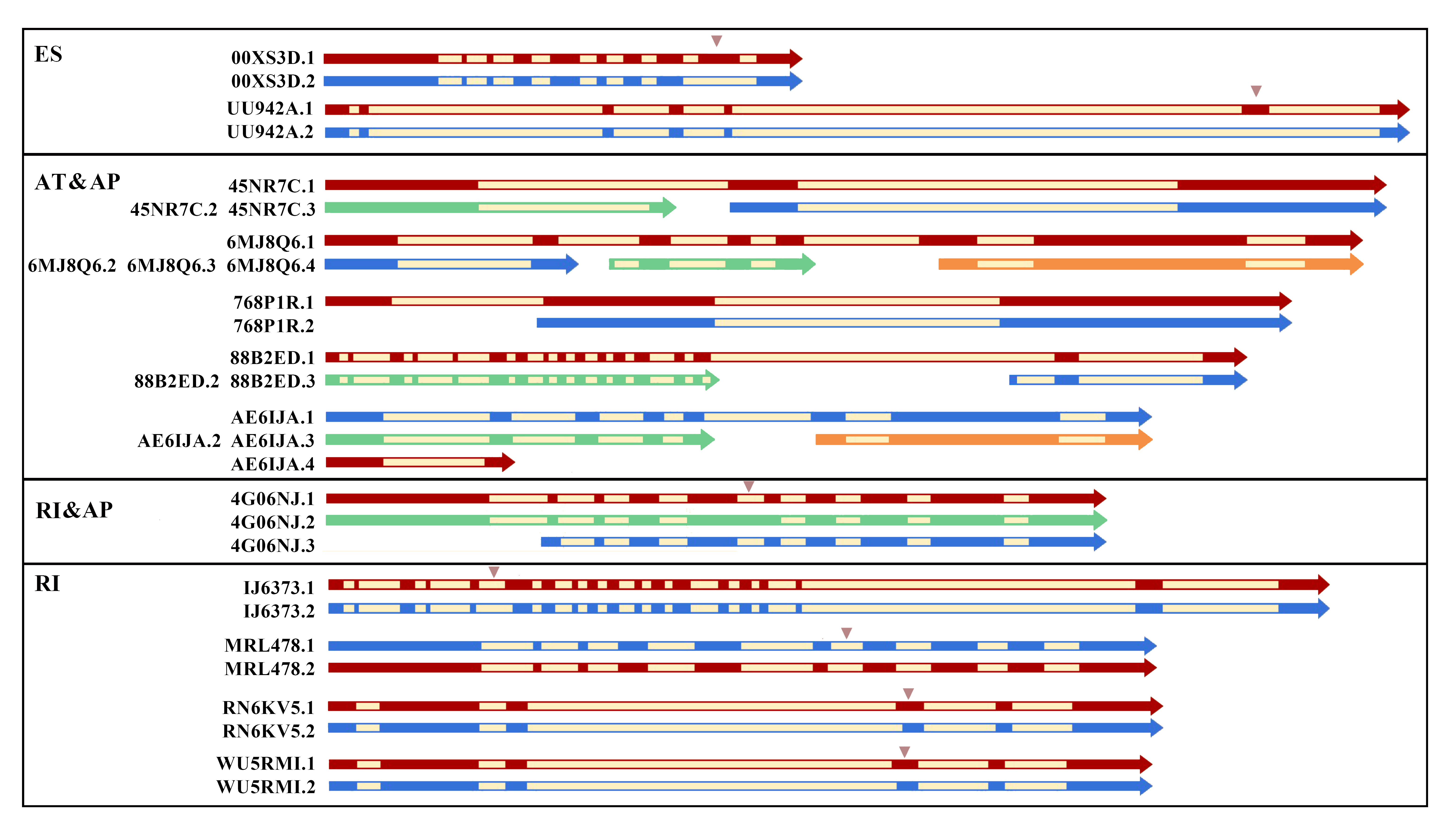

Supplement: Supplementary Figure 4 — Alternative splice (AS) analysis of Apetala2/ethylene-responsive factor (AP2/ERF) genes in A. hypogaea. ES, exon skip; AP, alternate promoter; AT, alternate terminator; RI, retained intron. The red inverted triangle indicated the difference of AS. [file Image_4.JPEG]

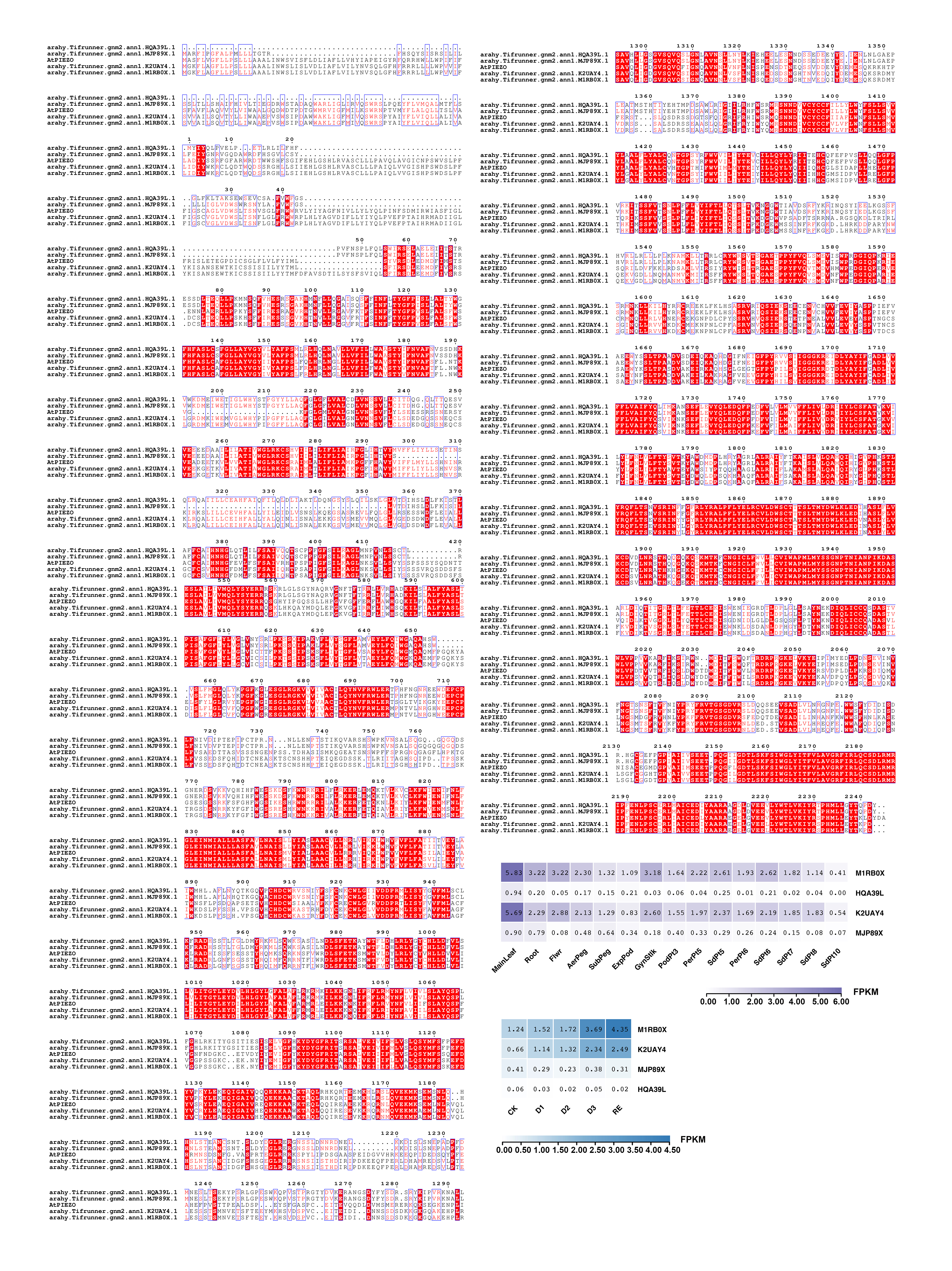

Supplement: Supplementary Figure 5 — Protein sequence alignment and express pattern of AhPiezo. [file Image_5.JPEG]
