# Supplementary material for: Genome-Wide Analysis and Expression Profiles of Ethylene Signal Genes and Apetala2/Ethylene-Responsive Factors in Peanut (Arachis hypogaea L.)
Source: Front Plant Sci. 2022 Mar 17;13:828482. doi: 10.3389/fpls.2022.828482 (PMC8968948; doi:10.3389/fpls.2022.828482)
Supplement: Supplementary Table 1 — The primers of quantitative PCR (qPCR). [file Table_1.DOCX]

**Supplementary Table 1** The primers of qPCR.

| Gene Name | Primer-F (5'-3') | Primer-R (5'-3') |
| --- | --- | --- |
| EIN3-J729H0 | CAACCTCTCCCTTGATTGGA | CAACCTCTCCCTTGATTGGA |
| EBF-G4JMEM | ACCCTTGTTGCTGATTGAGG | TGCAATTGCTGTTGGGACTA |
| ERF-4XS3FZ | TGCCGCTCAAAGAAGACGAT | TCTTCAATCGGGGCTACGAC |
| ERF-AS0C7C | GATCCAGGCAAGAAGAGCCG | AGGTCTAAGGGCGACGAATC |
| ERF-G0YQ1S | CCGCGGCAGTTAAGTTCAAG | AAGCATCCACATCGCCGTAA |
| ERF-ZW7540 | TGCCGCTCAAAGAAGACGAT | TCTTCAATCGGGGCTACGAC |
| ERF-QGFJ76 | AGCTGATTCGTCGCCCTTAG | ATTCTTAGCGACATCGCCGT |
| ERF-HGAZ7D | CTCCCAACGAAAACGCCAAC | CTCCCAACGAAAACGCCAAC |
| ELF1B-E3HYWR | AAGCTTCCCTGGCAAAGCTCAA | TTCCTCAGCTGCCTTCTTATCC |
